# Design and Development of Turbodrill Blade Used in Crystallized Section

**DOI:** 10.1155/2014/682963

**Published:** 2014-09-02

**Authors:** Wang Yu, Yao Jianyi, Li Zhijun

**Affiliations:** Key Laboratory on Deep Geo-Drilling Technology of the Ministry of Land and Resources, China University of Geosciences, Beijing 100083, China

## Abstract

Turbodrill is a type of hydraulic axial turbomachinery which has a multistage blade consisting of stators and rotors. In this paper, a turbodrill blade that can be applied in crystallized section under high temperature and pressure conditions is developed. On the basis of Euler equations, the law of energy transfer is analyzed and the output characteristics of turbodrill blade are proposed. Moreover, considering the properties of the layer and the bole-hole conditions, the radical size, the geometrical dimension, and the blade profile are optimized. A computational model of a single-stage blade is built on the *ANSYS CFD* into which the three-dimensional model of turbodrill is input. In light of the distribution law of the pressure and flow field, the functions of the turbodrill blade are improved and optimized. The turbodrill blade optimization model was verified based on laboratory experiments. The results show that the design meets the deep hard rock mineral exploration application and provides good references for further study.

## 1. Introduction

Turbodrill has been used in oil and gas industry over one century, yet it remains relatively obscure [1, 2]. As geothermal resources, oil and solid mineral [3] are mostly reserved at shallower depths and are now nearly depleted. The decline rate of producing reservoirs accelerates. The search for economically viable reservoirs in the world now focuses on drilling to greater depths. So the drilling technology under high temperature and high pressure (HTHP) conditions has become a hot topic in recent years. Deeper drilling in the crystallized rock formation has faced many challenges such as greater hardness [4], poor formation drillability [5], elevated temperatures [6], higher pressures, and higher costs [7]. All parts of turbodrill are heat-resistant because they are made of metal. Moreover, turbodrill is successful in hard and abrasive formations [8, 9], such as the crystallized rock, because of the compatibility with drill bit types used for drilling and coring these formations and the long life of turbodrills.

Turbodrill is a type of hydraulic axial turbomachinery which has a multistage blade consisting of stators and rotors. It converts the hydraulic power provided by the drilling fluid to mechanical power through turbine motor while diverting the fluid flow through the stator vanes to the rotor vanes [10]. The turbodrill blade is the heart of turbodrill, and its design and casting technologies are very important to the continual success of turbodrill [7]. Compared to the same specifications of PDM (positive displacement motor), the turbodrill rotation speed is usually significantly higher, which leads to the mismatch to the drill bit; the report [11] seeks to build on the successful high temperature turbodrills with additional speed reducer and adjustable bent housing, which are used to drill wells at Los Alamos National Laboratory's hot and dry rock project. Another research [12] introduces a more efficient 2-7/8 in-diameter turbodrill and a novel 4-1/8 in-diameter drill bit for drilling with coiled tubing. Some of the coiled turbine blades [13, 14] are designed on the basis of the requirements of the small diameter drilling. Jianhong et al. [15] calculated the pressure drawdown at the inlet/outlet and the pressure distributions in single-stage turbine for noncoring drilling. In this paper [10], we present computational fluid dynamics (CFD) simulations of a single-stage coiled tube turbodrill performance with different rotation speeds and mass flow rates [16] and then fluid-structural interaction (FSI) analyses for this small size turbodrill in which the finite element analyses of the stresses are performed based on the pressure distributions calculated from the* CFD* modeling [17]. The turbodrills are widely used in petroleum drilling, but mineral exploration drilling objectives and environment are quite different from petroleum drilling. The core drilling especially is very different from the noncoring drilling with the drilling parameters and drilling process which can lead to particular turbodrill blade.

The turbine drilling tool can be configured to match the application variables as required to optimize performance. Different types of blades can be used to produce different performances [7]. Unfortunately, all of these properties cannot be achieved simultaneously and the optimization always involves some degree of trade-off [18]. In this paper, the basic design methodology of coring turbodrills used in crystallized section is briefly covered. Also the numerical simulation approach for the turbodrill performance analysis is described. Then the simulation results are presented and discussed. At the end, the optimal turbodrill blade is manufactured and tested in laboratory.

## 2. Hydrodynamic Model of Turbodrill Blade

### 2.1. The Hypotheses of Model

**Figure 1:**
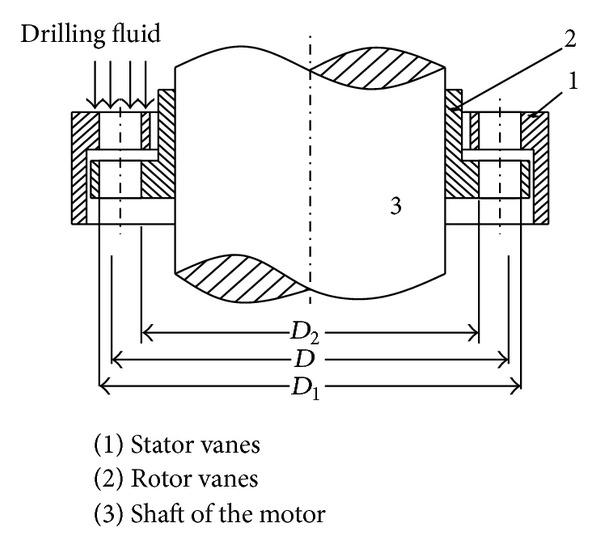
Sketch of the turbodrill blade assembly.

Turbodrill blade converts the hydraulic power provided by the drilling fluid to mechanical power through turbine motor while diverting the fluid flow through the stator vanes to the rotor vanes.  Figure 1 shows a typical turbodrill blade assembly and the drilling fluid flow path through turbine stage. The stator vanes are fixed by the axial preload force. Led by the stator vanes (1), the mud flows into the passages among turbodrill blades, leading the rotor (2) to the shaft of the motor which is connected to the bit. (3) The rotors are driven by fluid, providing the required torture and force.

The mud flows into the passages among turbodrill blades which can be regarded as different pieces of coaxial cylindrical movement. Different distances of each layer of liquid particles to the central axis result in different speed. There must be an average diameter (*D*) which has the average speed of turbine blade performance which is similar to considering all cylindrical layer flow movement performances of turbine blades. This method is called the unit theory method. So the average diameter is calculated as follows:

(1) $$\begin{array}{c} D=\frac{2\left(D_{1}^{3}-D_{2}^{3}\right)}{3\left(D_{1}^{2}-D_{2}^{2}\right)}. \end{array}$$

On the basis of Euler equations, the hydrodynamic model is built on four hypotheses: (1) the fluid is ideal; (2) turbodrill blade is infinitely thin; (3) turbodrill blade is countless; and (4) the flow and pressure losses can be neglected. So the fluid flow can actually move as expected without any friction.

### 2.2. The Model of Energy Transformation

**Figure 2:**
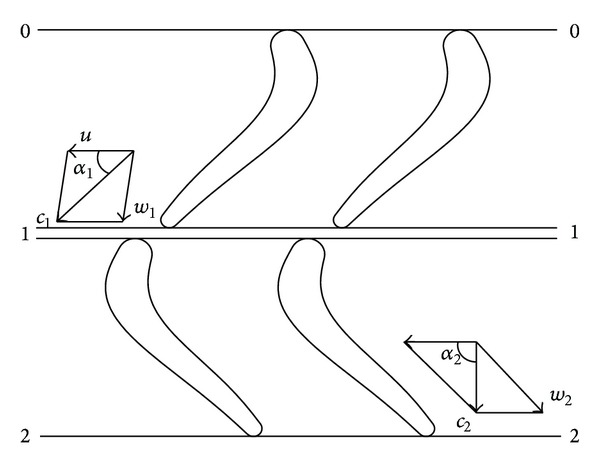
Turbine stage velocity diagrams.

Figure 2 shows the method of building velocity triangles when analyzing the turbodrill blade unit profile. This method is useful for visualizing changes in the magnitude and direction of the fluid flow due to its interaction with the blade system [3]. The two points 0, points 1, and points 2 along a streamline are the stator vanes input, stator vanes output, and rotor vanes output, respectively. The Bernoulli equation per unit weight between the three points can be obtained as

(2) $$\begin{array}{c} \frac{p_{0}}{\rho g}+\frac{c_{0}^{2}}{2g}+z_{0}=\frac{p_{1}}{\rho g}+\frac{c_{1}^{2}}{2g}+z_{1}+h_{s},\\ \frac{p_{1}}{\rho g}+\frac{c_{1}^{2}}{2g}+z_{1}=\frac{p_{2}}{\rho g}+\frac{c_{2}^{2}}{2g}+z_{2}+h_{r}. \end{array}$$

When the fluid flows through the stator vanes, there is no mechanical energy. The pressure energy is transformed into kinetic energy with partial hydraulic losses. However, most energy will be transformed into mechanical energy with partial hydraulic loss when the fluid goes into the rotors vanes.

The different height of drilling fluid can be ignored due to small values between the two sections. Because the flow through each level of the turbine blades is approximately equal and all levels of the length and the structures of the turbine blade are the same, there is no flow law. The flow rate between the turbines at the same level is also similar, meaning that *c*_2_ = *c*_0_. So the whole output mechanical energy of single-stage turbine blade is as follows:

(3) $$\begin{array}{c} H_{i}=\frac{p_{0}-p_{2}}{\rho g}-\left(h_{hs}+h_{hs}\right). \end{array}$$

In terms of the single-stage turbine blade, the torque transformed to the rotors is equal to the torque fluid received in different directions. Based on the law of moment of momentum, the output torque of single-stage rotors is calculated as

(4) $$\begin{array}{c} M_{i}=\frac{1}{2}\rho Q_{i}D\left(c_{1}\cos\alpha_{1}-c_{2}\cos\alpha_{2}\right)=\rho Q_{i}R\left(c_{1u}-c_{2u}\right). \end{array}$$

The transfer power consumption of single-stage blade is

(5) $$\begin{array}{c} N_{i}=M_{i}\omega =\rho Q_{i}R\omega \left(c_{1u}-c_{2u}\right)=\rho Q_{i}u\left(c_{1u}-c_{2u}\right). \end{array}$$

According to the energy conservation, the drilling fluid pressure energy can be transformed into mechanical energy. So the energy conversation formula can be expressed as

(6) $$\begin{array}{c} N_{i}=\rho gQ_{i}H_{i}=M_{i}\omega . \end{array}$$

The transformed mechanical energy of the pressure head is

(7) $$\begin{array}{c} H_{i}=\frac{u}{g}\left(c_{1u}-c_{2u}\right). \end{array}$$

The turbodrill blade is a series of the connections in the shell of the turbodrill. Suppose that *Z* is the numbers of the multistage turbodrill blades. The output torque of single-stage rotors, the energy, and the pressure head are calculated as follows:

(8) Mi=ZρQiR(c1u−c2u),Ni=ZρQiu(c1u−c2u),Hi=Zug(c1u−c2u).

### 2.3. The Design Model of Turbodrill Blade

The output mechanical energy of turbodrill is related to the numbers of blades stages, radius size, and blade profile. However, the feasible method is increasing the different value of the circumferential speed because of uncomfortable use of the turbine when enlarging the length and radius size of the turbodrill. So it is important to optimize the profile, structure angle, and solidity of the turbodrill blade.

**Figure 3:**
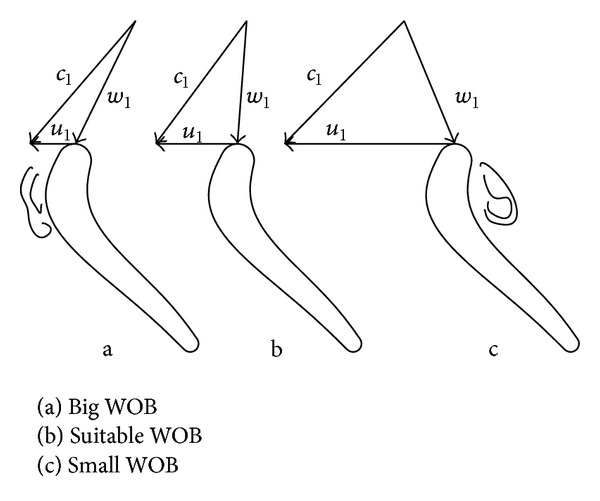
Hydraulic loss occurring on different WOB.

The field conditions must be taken into account when optimizing the turbodrill blade. The value of the circumferential speed of the turbodrill blade changes with the variation of the weight on bit (WOB).  Figure 3 displays that a hydraulic loss will occur on the back of the blade exerted when the WOB is big, while the flow separation loss will occur on the basin of the blade when the WOB is small. In order to avoid the loss caused by the impact of fluid, the inlet flow angle (*β*_1_) should be equal to the inlet structure angle (*β*_1*k*_) in stators blade. *α*_2_ = *α*_2*k*_ is also satisfied in rotors blade, in which working condition is called the no-impact status.

Three dimensionless parameters, such as the axial velocity factor $\left(\overset{-}{c_{z}}\right)$, impact coefficient (*m*_*a*_), and circulation flow coefficient $\left(\overset{-}{c_{u}}\right)$, are selected to design and optimize the turbodrill blade profile with fixed size of the stage number and radius size:

(9) cz¯=czu0=60Qiπ2D2bφn0=1cot α1k+cot β1k =1cot α2k+cot β2k,cz=QiπDbφ,u0=πDn060.

The axial velocity factor is the ratio between the axial flow velocity (*c*_*z*_) and the circumferential speed of the blade (*u*_0_), which is related to the blade structure angle. The axial velocity factor is established as (9).

The impact coefficient, which is the ratio of the kinetic energy transformed from the pressure head, is affected only by the blade structure angle and is presented as

(10) $$\begin{array}{c} m_{a}=\frac{1}{H_{i}}\left(\frac{c_{1}^{2}-c_{2}^{2}}{2g}\right)=\frac{\text{cot}\;\alpha_{1k}+\text{cot}\;\alpha_{2k}}{2\left(\text{cot}\;\alpha_{1k}+\text{cot}\;\beta_{1k}\right)}. \end{array}$$

The circulation flow coefficient describes the ratio between the dynamic factor and the kinematics indexes; it is affected only by the blade structure angle and is presented as

(11) $$\begin{array}{c} \bar{c_{u}}=\frac{c_{1u}-c_{2u}}{u_{0}}=\frac{\text{cot}\;\alpha_{1k}-\text{cot}\;\alpha_{2k}}{\text{cot}\;\alpha_{1k}+\text{cot}\;\beta_{1k}}. \end{array}$$

Based on the axial velocity factor, the impact coefficient, the circulation flow coefficient, and the structural angle can be deduced as

(12) cot α1k=c1ucz=1cz¯(ma+cu¯2),cot α2k=c2ucz=1cz¯(ma−cu¯2),cot β1k=ω1ucz=1cz¯[1−(ma+cu¯2)],cot β2k=ω2ucz=1cz¯[1−(ma−cu¯2)].

The minimum hydraulic loss occurs when there is no impact on the turbodrill blade. By changing the first derivative differential Euler equation, the output torque, the bit rotation speed, the turbodrill blade pressure drop, and the running-in speed can be calculated as

(13) M0=ηv2ηm2πbφρQ2cu¯cz,n0=60ηv(πD)2bφQcz¯,H0=(ηvπDbφ)2ρQ2ηhcu¯cz−2,nx=n0·(1+cu¯).

The equation shows that the output torque under the premium working condition is proportional to circulation flow coefficient. The bit rotation speed is only opposite to the axial velocity factor. The specialized function of the turbodrill blade can be translated by adjusting the axial velocity factor and circulation flow coefficient. When the hydraulic efficiency (*η*_*h*_) is increasing, the pressure drop will be reducing simultaneously. The hydraulic efficiency is also related to the axial velocity and circulation flow coefficient. However, considering the hydraulic efficiency, it is recommended that the axial velocity factor and circulation flow coefficient should be adjusted within proper domain.

## 3. Design of Turbodrill Blade

### 3.1. The Design Goal and Diagram

The turbodrill is designed to satisfy the coring in the crystalline rock formations, whose drillability classification number is from 7th to 8th. The parameters of the turbodrill blade should be designed as in Table 1.

**Figure 4:**
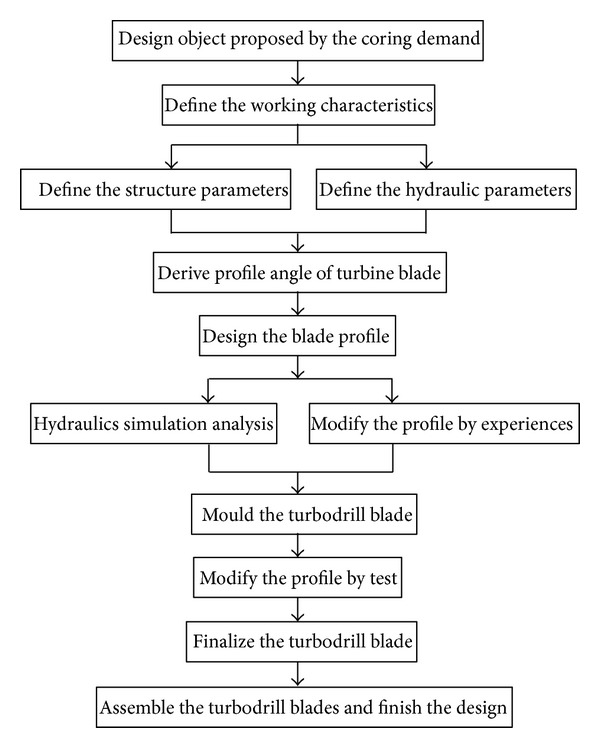
The design diagram for turbodrill blade.

The turbine blade design includes the parameters of the working characteristics, the optimum radius, the axial dimension, and the profile and the parameters of the blade structures. The design diagram for the turbodrill blade is shown in Figure 4. First, the working characteristics, such as the rated output torque, the optimal rotation speed, and the pressure drop, are defined by the coring demand in the crystalline rock formations.

The outer diameter Φ 127 mm turbodrill is generally equipped with Φ 152 mm impregnated diamond bit. 2–4 rpm of linear velocity ensures a higher rock-crushing efficiency. The range of 250–500 rpm is regarded as the optimal velocity after our calculation, demanding that the blade structure should be adjusted to lower the speed. Second, after the structure parameters are preliminary determined, such as the radius and the axial dimension, the parameters of blade structure angles can be calculated under the no-impact status according to three precedent dimensionless parameters. So the turbodrill profiles are drawn out. Third, they are optimized repeatedly by the simulations, the experience, and the laboratory experiments until the output performances of turbodrill are satisfied.

### 3.2. Structure Design of Turbodrill Blade

Except for the profile, the outer cylinder size, the radius size, and the axial dimension, other parts are also included in the design. The calibration and material selection are also accomplished in this section. The outer diameter of the tube and turbine stator should be increased as big as possible according to the borehole and annular clearance. The material of the turbodrill tube is 40CrMo alloy steel which has high mechanical properties and good performances of heat treatment for petroleum drilling.

The solid rotor shaft, which bears the positive torque from the rotors and antitorque from the bit, should be as slim as possible to meet sufficient stress intensity. The relationship between the diameter of the rotor shaft and the allowable twisting stress is shown as in (14). The diameter of the rotor shaft is equal to the inner diameter of the rotor with a clearance fit, so the inner diameter of the turbine rotor is decided by the rotor shaft:

(14) $$\begin{array}{c} d\geq \sqrt[{3}]{\frac{ST}{\left[\tau \right]}}. \end{array}$$

**Figure 5:**
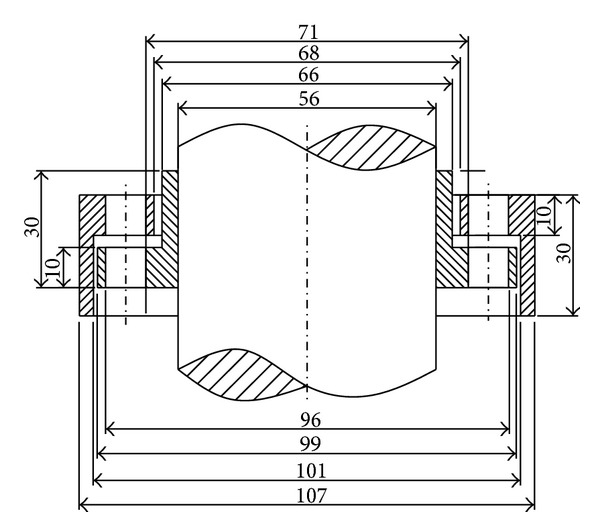
Part of the design dimensions.

In order to avoid the interference between stator vanes and rotor vanes, a clearance should be retained. Considering the precision machining limit, the clearances between the static and dynamic component parts are about 1 to 2 mm. According to this design principle, the parts of the design dimensions are shown in Figure 5.

When the volumetric efficiency *η*_*v*_ is 0.9 and the reduction coefficient of the blade *φ* is 0.9, the axial component of the absolute flow velocity is calculated as

(15) $$\begin{array}{c} C_{1z}=\frac{Q\cdot \eta_{v}}{\pi Db\varphi }=\left(10\sim 15\right)\cdot 0.287\;\text{m}/\text{s}=\left(2.87\sim 4.315\right)\;\text{m}/\text{s}. \end{array}$$

### 3.3. Profile Design of Turbodrill Blade

**Figure 6:**
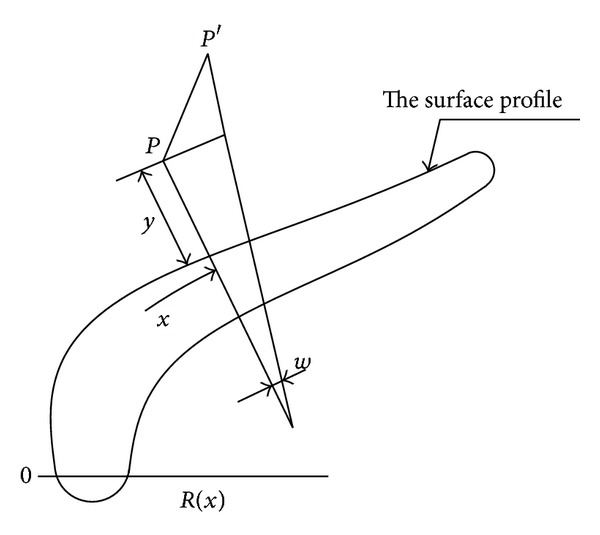
The 2D coordination of the turbodrill blade boundary.

The blade runner field is changed mainly by influencing the speed and pressure of the flow near the blade surface. In order to keep a higher efficiency, the speed and pressure should be changed smoothly, reducing the fluid friction, flow separation loss, and blade end pressure loss. Since then, 2D orthogonal curvilinear coordinates are established to research the influence of the blade line on the law of the fluid move on the blade surface. The fluid field can be changed by affecting its speed and pressure. That smooth speed and the pressure distribution ensure high efficiency.  Figure 6 displays a 2D coordinate of the turbodrill blade boundary. On the coordinates of the point *P*(*x*, *y*), *x* is the dorsal arc along the surface profile from the origin (O), and *y* is the vertical distance from the normal line of the surface profile. *u* and *v* are the corresponding speeds of the two directions, respectively.

On the basis of the primary motion equation about the steady flow boundary of the incompressible fluid, the massive conservation and momentum conservation can be expressed as

(16) ∂u∂x+∂∂x[(1+yR)v]=0,Ru∂u(R+y)∂x+v∂u∂y+uvR+y=−R∂P(R+y)ρ∂x +v[−R∂2v(R+y)∂x∂y+∂2u∂y2 +∂u(R+y)∂y+R∂v(R+y)∂x−u(R+y)2],Ru∂v(R+y)∂x+v∂v∂y−u2R+y=−∂Pρ∂y +v[−R∂2u(R+y)∂x∂y−R∂u(R+y)2∂x+R2∂2v(R+y)2∂x2 +R(R+y)2dRdx[uR+y+y∂u(R+y)∂x]].

The equations indicate that boundary curvature radius (*R*) influences the fluid parameter most. A continuous derivative of curvature can ensure smooth distribution. Suppose that the profile of the turbodrill blade is *y* = *f*(*x*) and its curvature and third derivative are, respectively,

(17) $$\begin{array}{c} C=\frac{1}{R}=\frac{y^{\prime \prime }}{{\left[1+{\left(y^{\prime }\right)}^{2}\right]}^{3/2}}=\frac{f^{\prime \prime }}{{\left[1+{\left(f^{\prime }\right)}^{2}\right]}^{3/2}},\\ C=\frac{f^{\prime \prime \prime }\left[1+{\left(f^{\prime }\right)}^{2}\right]-3f^{\prime }{\left(f^{\prime \prime }\right)}^{2}}{{\left[1+{\left(f^{\prime }\right)}^{2}\right]}^{5/2}}. \end{array}$$

Although combined profile can meet continuous shrinkage, it has no continuous curvature derivative. This can cause sudden change of the fluid speed and pressure and damage hydraulic property of the turbodrill. As a result, we use quintic polynomial combined with computer-aided design to develop profile of the turbodrill. Suppose that the pressure side and negative pressure side of the blade are *y*_*p*_ = *f*(*x*) and *y*_*s*_ = *g*(*x*). Consider

(18) yp=a0+a1x+a2x2+a3x3+a4x4+a5x5,ys=b0+b1x+b2x2+b3x3+b4x4+b5x5.

Based on the angles of the inlet and outlet, the four special points can be calculated. Put the four special points, first derivative, and second derivative into their equations, and the pressure side and negative pressure side of the blade can be described as

(19) [1xp1xp12xp13xp14xp151xpnxpn2xpn3xpn4xpn5012xp13xp124xp135xp13012xpn3xpn24xpn35xpn30026xp112xp1220xp130026xpn12xpn220xpn3][a0a1a2a3a4a5] =[yp1ypnyp1′ypn′yp1′′ypn′′],

(20) [1xs1xs12xs13xs14xs151xsnxsn2xsn3xsn4xsn5012xs13xs124xs135xs14012xsn3xsn24xsn25xsn20026xs112xs1220xs130026xsn12xsn220xsn3][b0b1b2b3b4b5] =[ys1ysnys1′ysn′ys1′′ysn′′].

**Figure 7:**
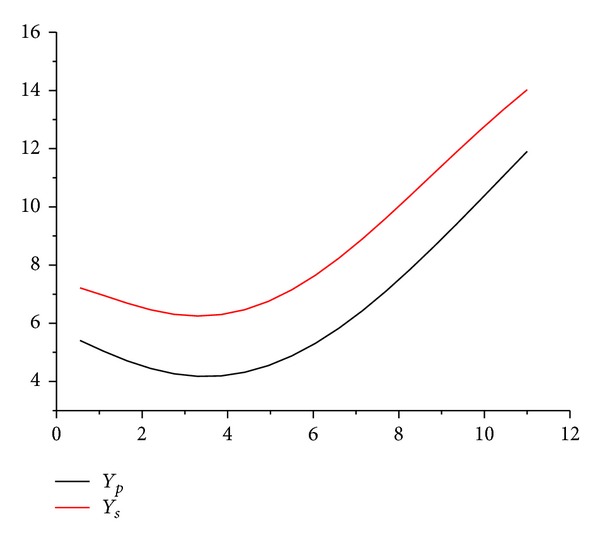
The preliminary profile of the blade.

The parameters of  *a*_0_, *a*_1_, *a*_2_, *a*_3_, *a*_4_, *a*_5_, *b*_0_, *b*_1_, *b*_2_, *b*_3_, *b*_4_, and *b*_5_ can be solved by the two linear equations (19) and (20).  Figure 7 displays the profile of the pressure side and negative pressure side of the blade when the output rotation speed *n*_0_ = 500 rpm and output torque *M*_0_ = 1000 Nm which are given by the corresponding relationship between torque and speed.

After checking the blade line, it shows that there are no reflection points on pressure surface and suction surface. This constitutes a continuous flow path contraction. It proves that the line has a relatively flow loss, meeting all the design requirements. Finally, according to the axial length of size constraints, a leading edge circle and a trailing edge circle are established on its tangent line at both ends of the blade so as to make the blade closed.

## 4. CFD Simulation of Turbodrill Blade

### 4.1. CFD Model

**Figure 8:**
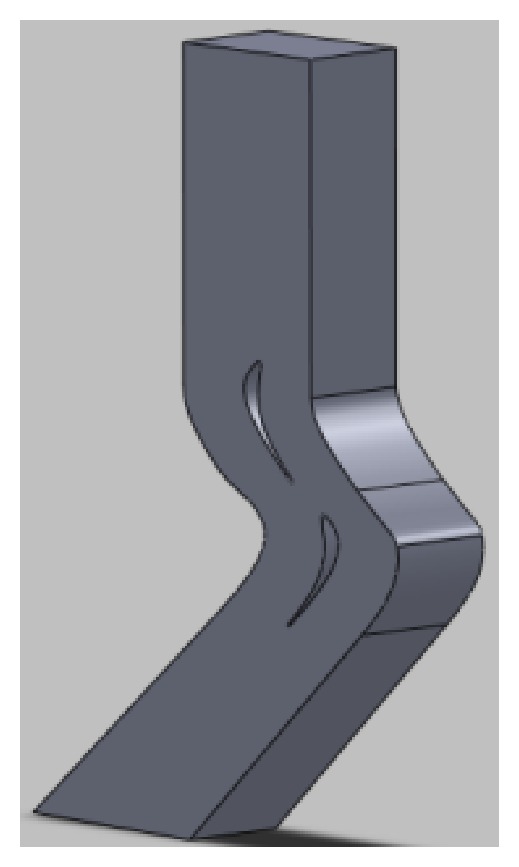
The single-period model in SolidWorks.

Computational fluid dynamics (CFD) has emerged as an effective optimization tool for the experiments because of its diverse applications in industry [19, 20]. Drawing with the* SolidWorks* a single cycle turbine blade airfoil-dimensional model (Figure 8), we can establish the flow field width on the basis of blade profile in two-dimensional model, extend three times of upper and lower widths to obtain import and export flow borders, and form a closed fluid channel; it is easy to get the stable solution of flow field and establish a single cycle cross-flow model, and we can establish complete three-dimensional models in SolidWorks formed in* ANSYS CFD* simulation model.

### 4.2. Meshing and Boundary Conditions

**Figure 9:**
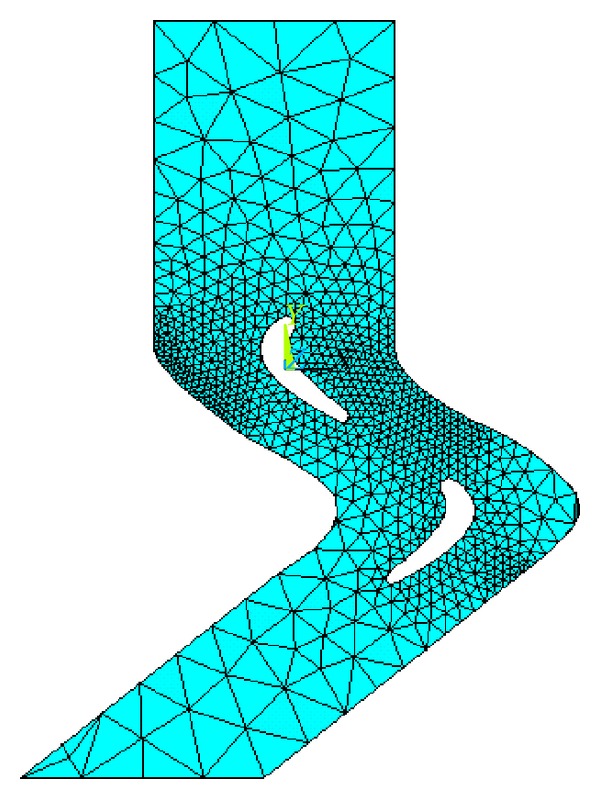
The* ANSYS* model of the turbodrill blade.

The blade is characterized by irregular shape, demanding an automatic mesh generation. In this simulation, a minimum unit meshing by free division ensures accurate results. There are 142 units meshing the finite element model in Figure 9. Although significantly greater than the extension of the grid around the blade grid, the meshes near the blade are relatively uniform. This proves that the mesh quality is high.* ANSYS* is used to research the differences between the flow fields. The curve of the blade is complex—short runner and relatively fast flow rate. After calculating Reynolds (Reynolds number), it proves to be turbulent. Different models need to set different solution control and execution control. Because this three-dimensional simulation of flow field is continuously differentiable turbulent flow, the calculation of control theory should be turbulence model theory. The default equation is Reynolds averaged equations. For unsteady turbulent flow,* ANSYS *analysis cites false concept of homeostasis without regard to the circumferential direction of the flow field changes, so at this mode of analysis it should be* TRAN* steady flow. The input flow and output flow are stable when the blades are applied, resulting in flow between the stator and the rotor blades relatively stable.

For the drilling fluid of the turbine blade, with certain density and viscosity, we can select fluid and then analyze and define these two worthy constants when we set fluid conditions. Other parameters generally use the defaults of* ANSYS* program system. After completing the relevant definitions and settings, we use Run Flotran to solve the corresponding analysis of the results. In the* ANSYS* postprocessor tune the corresponding calculation results are obtained using the* PLOT* function blade flow velocity and pressure fields for analysis.

### 4.3. Simulation Results

**Figure 10:**
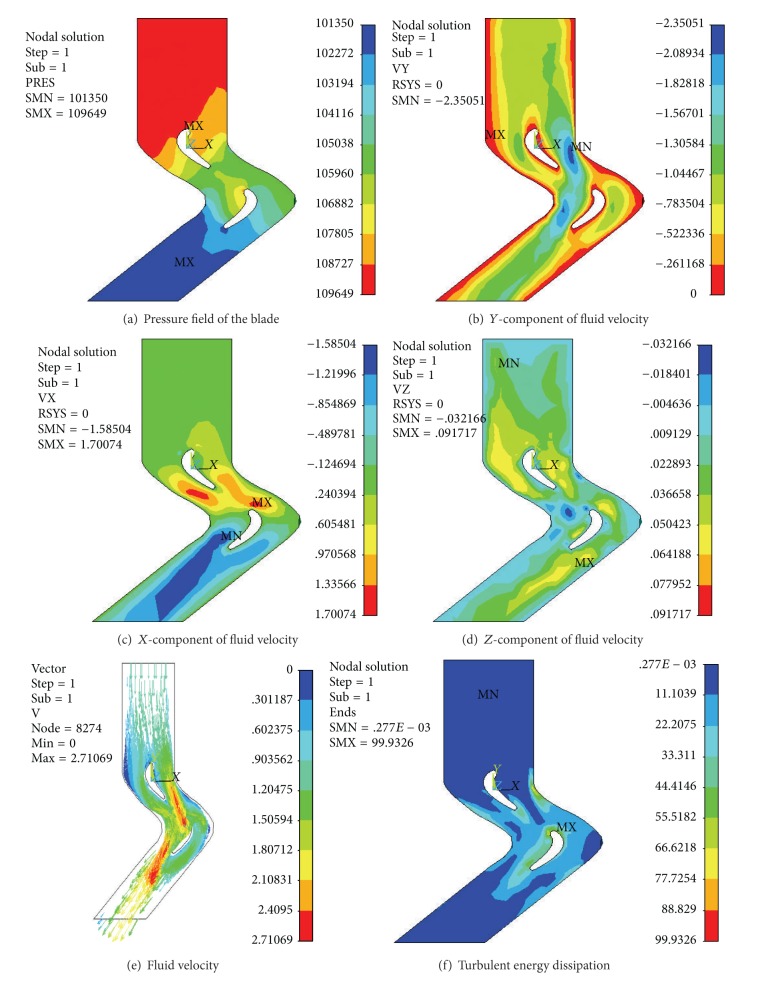
The flow field of I-type blade.

**Figure 11:**
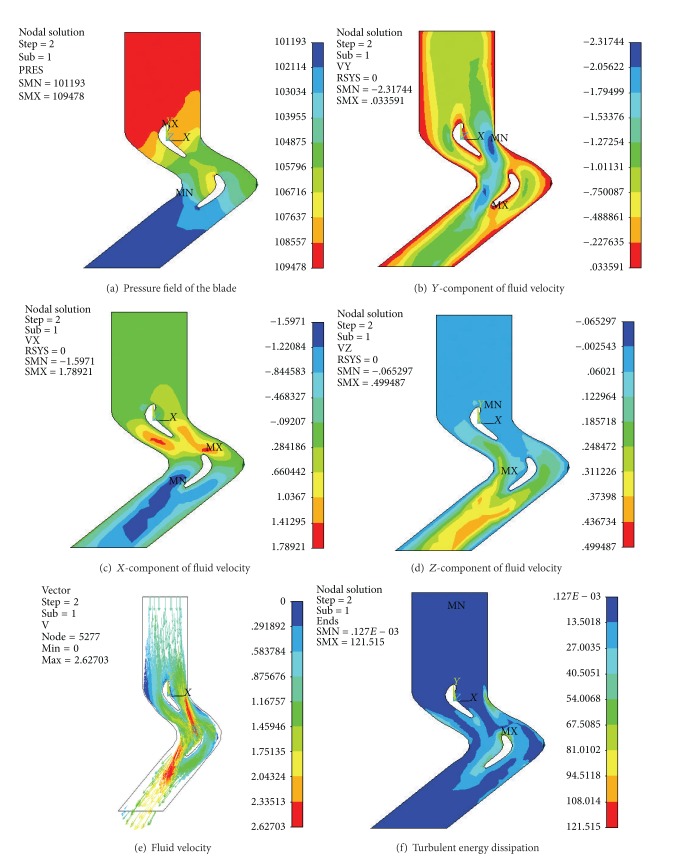
The flow field of II-type blade.

In order to select an optimum blade shape and verify its effectiveness, the* CFD* analysis of the two different turbine blades on the basis of the preliminary profiles was completed for low speed high torque flow field simulation. Figures 10 and 11 display the simulation results, including the pressure field of the blade, *Y*-component of fluid velocity, *X*-component of fluid velocity, *Z*-component of fluid velocity, fluid velocity, and turbulent energy dissipation.

The flow field simulation figure shows the following. (1) The *Y*-component and *X*-component of fluid velocity are the drilling fluid dominant orientation. These two kinds of fluid velocity become larger smoothly and gradually, which proves the reasonable design of the surface profile. (2) The *Z*-component of fluid velocity of both types of blade is very small, which shows the little energy dissipation. The *Z*-component of fluid velocity of the I-type blade is smaller than the II-type blade, which indicates that the former has less energy dissipation. (3) The drilling fluid flows into the turbine blades of the stator. The fluid impacts pressure surface and the suction surface after flowing into the blade. Then it will accelerate along the flow channel. The rotor work flow and pressure energy can be translated into kinetic energy of the rotor mechanical kinetic energy. (4) The pressure field surface pressure distribution from the inlet to the outlet ends in smooth descending order. The turning point of the leading edge of the blade is greater than the blade suction, from the description of the pressure gradient of the output torque of the size. The greater pressure gradient of the I-type leaf blade shows that it has more torque gradient than the II-type blade. (5) The small turbulent energy dissipation conforms to the *Z*-component of fluid velocity. The turbulent energy dissipation of the I-type blade is smaller than the II-type blade, which indicates that the former is better. In summary, the overall smooth flow and the low eddy current loss prove that both types of turbine blades correction are feasible. The results meet all the requirements. After careful comparative analysis, the I-type blade turns out to be more reasonable and suitable to manufacture the physical prototype for test.

## 5. Test of the Turbodrill Stage

### 5.1. Turbodrill Blade Casting

**Figure 12:**
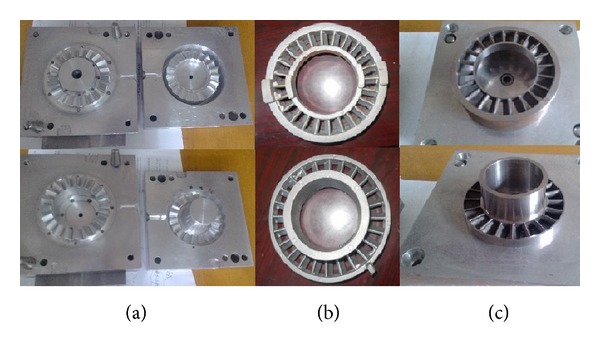
The casting process of the turbodrill blade.

Turbodrill blade is processed using fine casting molding. First, based on the blade structure and its curvature, a fine model (Figure 12(a)) is processed by CNC. Second, a vice blade blank should be modeled by pouring wax, shown in Figure 12(b). After the mating surface is processed, a blade can be made (Figure 12(c)).

### 5.2. Laboratory Test of the Turbodrill Blade

**Figure 13:**
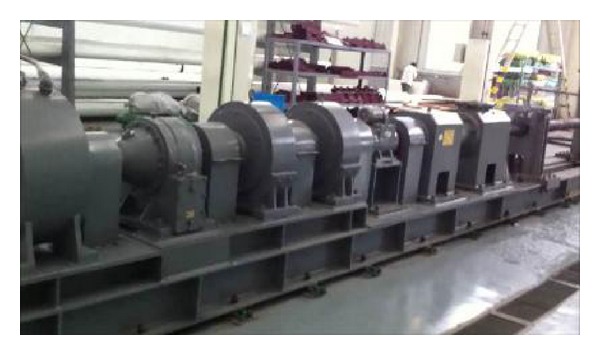
Laboratory test and its equipment.

The turbodrill prototype is composed of two turbine sections, which has one hundred turbine blades. In order to verify the effect of the turbodrill coring blade design, the single turbine section was tested on the drill test bench in Tianjin Dagang mechanical manufacturing companies on July 14, 2013. As shown in Figure 13, the test rig is mainly composed of the host, load system, circulatory system, and measurement system [21]. Experimental principle is as follows: the volume flowing into the turbine drill traffic is kept to a given value under the premise of the drill through the turbine power output of the torque applied to the different loads, so that drilling in different stable braking torque works. It can test output torque, output speed, and pressure loss under different conditions. Also, it can be used to research the relationship among the output torque, efficiency of the turbine drill, and the rotational speed.

**Figure 14:**
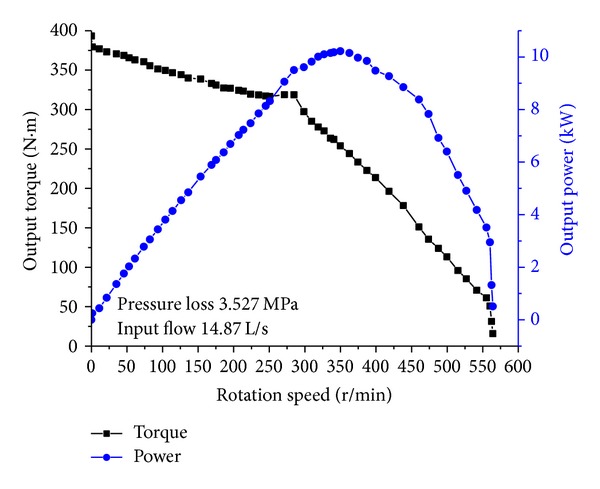
Laboratory test results.

Figure 14 shows the output torque and power variation with the rotational speed. By using the water (1 g/cm^3^) as the drilling fluid, the maximal output torque and maximal rotational speed of the single turbine section are 394 N*·*m and 564 rpm, respectively. Considering the actual density of the drilling fluid is the 1.25–1.3 times of water and that the output torque is proportional to the density of the drilling fluid, the maximal output torque of the turbodrill prototype with two turbine sections can reach up to 985–1025 N*·*m, which is equal to the design goal. With the increase of the drilling fluid, the rotational speed will decrease slightly. The test results are equal to the design goals, which can verify the accuracy in surface profile indirectly.

## 6. Conclusions

1. Considering high pressure and temperature, hydraulic units with high torque and low speed are applied. The turbodrill is developed with optimized parameters. The surface profiles of the turbodrill are designed on the basis of dimensionless coefficients.
2. The effect of the blade profile's change on the first curvature is figured out: the first curvature of the blade front virtually affects the blade front end. Lower curvature leads to higher front and smoother flat. First curvature of the blade back virtually affects the blade back end. Lower curvature leads to higher turbodrill blade profile.
3. The single-period model is developed through* ANSYS CFD*. The effects of different fluid discharge and viscosity on hydraulic property are performed. The optimized design fitting curve is confirmed.
4. The basic design methodology and method of coring turbodrills used in crystallized section are efficacious. The results show that the design meets the deep hard rocks mineral exploration application and provides good references for further study.

## Figures and Tables

**Table 1:** Target performance parameters of turbodrill (*Ф*127).

Items/unit	Value
Outside diameter/mm	127
Working flow/L*·*s^−1^	10~15
Rotation speed/r*·*min^−1^	200~500
Pressure drop/MPa	≤5
Rated torque/N*·*m	800~1200
Drilling fluid density/kg*·*m^−3^	1000~2000

## Nomenclature

*D*: Average diameter of drilling fluid
*D*_1_: The maximum diameter of drilling fluid
*D*_2_: The minimum diameter of drilling fluid
*P*_0_: Hydraulic pressure of drilling fluid in stators import
*P*_1_: Hydraulic pressure of drilling fluid in stators export
*P*_2_: Hydraulic pressure of drilling fluid in rotors import
*c*_0_: Absolute velocity of drilling fluid in stators import
*c*_1_: Absolute velocity of drilling fluid in stators export
*c*_2_: Absolute velocity of drilling fluid in rotors import
*z*_0_: Elevation head of drilling fluid in stators import from the datum line
*z*_1_: Elevation head of drilling fluid in stators export from the datum line
*z*_2_: Elevation head of drilling fluid in rotors import from the datum line
*h*_*s*_: Energy losses per unit weight of drilling fluid from section 0-0 to section 1-1
*h*_*r*_: Energy losses per unit weight of drilling fluid from section 1-1 to section 2-2
*ρ*: The density of the drilling fluid
*H*_*i*_: The whole output mechanical energy head of single-stage turbine blade
*M*_*i*_: The output torque of single-stage rotors
*Q*_*i*_: The flow of the drilling fluid
*D*: The inner diameter of the turbodrill
*R*: The inner radius of the turbodrill
*α*_1_, *α*_2_: The blade exit angle of the stator vane and rotor vane
*N*_*i*_: The transfer power consumption of single-stage blade
*ω*: The angular velocity of the output shaft
*Z*: The numbers of the multistage turbodrill blades
*α*_2_, *β*_1_: The inlet flow angle in rotors blade and stators blade, respectively
*α*_2*k*_, *β*_1*k*_: The inlet structure angle in rotors blade and stators blade, respectively
*c*_*z*_: The axial flow velocity
*u*_0_: The circumferential speed of the blade
*φ*: The reduction coefficient of the blade
*t*: The pitch of the solidity
*Q*_*i*_: The flow in the turbodrill blade
*B*: The radial height of the turbodrill blade
*n*_0_: Rotational speed at the suitable WOB
*ψ*: The outlet flow angle
*δ*: The blade thickness on outlet
*H*_*i*_: The pressure head
*c*_1_, *c*_2_: The inlet flow absolute speed of the rotor and stator blade
*c*_1*u*_, *c*_2*u*_: Circumferential component of the inlet flow absolute speed
*d*: The diameter of rotor shaft
*T*: The torque on the rotors shaft
[*S*]: Safety factor of the shaft materials
[*τ*]: The allowable twisting stress
*R*: The radius of curvature
*V*: Kinematic viscosity
*P*: Pressure.
